# Recent Advancement in Boron-Based Efficient and Pure Blue Thermally Activated Delayed Fluorescence Materials for Organic Light-Emitting Diodes

**DOI:** 10.3389/fchem.2020.00373

**Published:** 2020-05-19

**Authors:** Hyuna Lee, Durai Karthik, Raju Lampande, Jae Hong Ryu, Jang Hyuk Kwon

**Affiliations:** Organic Optoelectronic Device Laboratory, Information Display Department, Kyung Hee University, Seoul, South Korea

**Keywords:** boron acceptor, thermally activated delayed fluorescence (TADF), multiple resonance, electroluminescence, blue organic light-emitting diode (OLED)

## Abstract

In the last few years, electron-deficient materials have been actively researched for application in organic light-emitting diode (OLED) as dopant and electron-transporting materials. The boron-containing materials are interesting as they give good emissive properties in solid state with an electron-accepting character. Recently, many boron-containing materials are used as emissive materials for thermally activated delayed fluorescence (TADF) OLED applications. In this review, boron acceptor-based push–pull small molecules used for application in blue TADF OLEDs are reviewed, covering their different types of acceptor, molecular design, structure–property relation, material properties, and device properties. Also, the importance of boron acceptors to address the key issue of blue TADF OLEDs is discussed.

## Introduction

Organic light-emitting diodes (OLEDs) have been actively investigated in the last three decades since the first discovery by Tang and Vanslyke (1987) because of their incomparable advantages as they offer low power consumption, high brightness, color purity, light weight, large viewing angle, and flexible nature compared to other lighting sources. The first-generation fluorescent OLEDs exhibited lower efficiency due to usage of only 25% of singlet excitons formed during the exciton recombination process. Later, second-generation OLEDs were developed to utilize both singlet and triplet excitons to increase the internal quantum efficiency (IQE) up to 100% using heavy metal atoms, which are also called phosphorescent OLEDs (Baldo et al., 1998). Though the efficiency, color purity, and device lifetime of phosphorescent green and red OLEDs are satisfactory to commercialize, blue phosphorescent OLEDs suffer from low efficiency, poor color purity, and short device lifetime due to high triplet energy of blue emitters (Scholz et al., 2015; Im et al., 2017). Later, third-generation OLEDs were demonstrated with 100% IQE by using the concept of thermally activated delayed fluorescence (TADF) process in pure organic materials (Uoyama et al., 2012). These TADF materials are promising to achieve high efficiency and color purity in the blue OLEDs. Thus, recently, many reports were published on blue TADF emitters based on different types of donor and acceptor design concepts (Liang et al., 2019).

**Figure 1:**
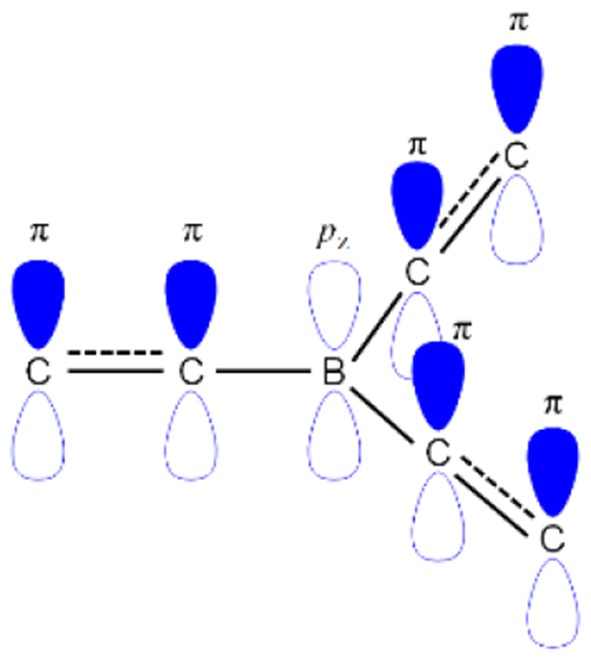
General configuration of *p*_*z*_-π conjugation of B–C atoms in the organic conjugated systems.

The boron-based materials have received tremendous interest in the last few years as they are widely used as potential candidates in the optoelectronic devices (Entwistle and Marder, 2004; Turkoglu et al., 2017; Mellerup and Wang, 2019). Boron atom has a vacant p-orbital which gives an electron-deficient nature or Lewis acidic nature (Brown and Dodson, 1957). This electron-deficient boron can make a π-conjugation with organic conjugated system through empty *p*_z_ orbital of boron and π-orbital of carbon as shown in Figure 1. Recently, boron-based materials have drawn immense attraction in designing as new TADF acceptor moieties because they offer excellent photophysical and electrochemical properties. Especially, boron materials show high photoluminescence quantum yield (PLQY) because of their sp^2^ hybridized trigonal planar geometry which gives rigid/planar molecular structure resulting in less non-radiative decay (Yamaguchi and Wakamiya, 2006; Elbing and Bazan, 2008; Von Grotthuss et al., 2018). Generally, boron-containing materials exhibit largely blue emission due to their weak acceptor nature, which yields a large band gap. Though the boron atom has a weak acceptor nature, boron acceptor materials have exhibited different color emissions varying from blue to red depending on the acceptor design strategy (D'aléo et al., 2017; Yang et al., 2018; Zhang et al., 2019). Not only the tri-coordinate boron shows excellent emission properties, but the tetra-coordinate boron also shows very good emissive properties as evident from BODIPY (boron-dipyrromethene) materials (Bonardi et al., 2008; Schellhammer et al., 2017; Stachelek et al., 2017; Zampetti et al., 2017). Unlike tri-coordinate boron, one important thing in tetra-coordinate boron is to keep the charge neutral of overall material by forming a covalent bond with mono-anionic chelate ligand to dissipate boron's negative charge. Therefore, the rigidity of the molecule is enhanced by virtue of chelation effect resulting in good emissive properties (Frath et al., 2014).

Recently, many TADF materials with boron acceptors have been reported with good performances. There are several boron TADF materials available in the literature for different color OLEDs. However, blue TADF OLEDs are interesting as they are promising alternatives to commercial fluorescent blue emitters. As boron-based blue TADF materials are promising to realize high efficiency with a long lifetime, the detailed knowledge on the structure–property relation and design tactics of boron-based blue TADF emitters is highly desirable. Thus, in this review, we focus on the boron acceptor-based materials used for blue TADF OLEDs. Also, we discuss the requirements of blue emitters and different types of boron acceptors (tri- and tert-coordinate boron) used for blue TADF materials. In addition, we also discuss multiple resonance TADF materials and their underlying mechanism which is particularly for boron blue TADF OLEDs. Finally, the future outlook summarizes to achieve high efficiency and long lifetime for boron-based blue TADF OLEDs.

## Basic Requirement of Blue Materials

Over the last few years, huge progress has been made on the synthesis of new blue TADF emitters for OLED applications. To use blue TADF material for OLED applications that should be capable of making high external quantum efficiency (EQE), low-efficiency roll-off, good color purity of x <0.15, y <0.10, and a long lifetime. To make OLED containing these properties, blue TADF material should have a high PLQY, wide energy gap (ΔE_ST_) between highest occupied molecular orbital (HOMO) and lowest unoccupied molecular orbital (LUMO), small ΔE_ST_ between the lowest singlet and triplet states, fast reverse intersystem crossing (RISC) from triplet to singlet, narrow full width at half maximum (FWHM), and high horizontal dipole orientation in the film.

Normally, small ΔE_ST_ can be achieved by reducing an exchange integral between HOMO and LUMO and a large twist angle between the donor and the acceptor. The use of a rigid and symmetric structure supports to enhance PLQY and to achieve a narrow FWHM. The high bandgap (S_1_) energy can be reached by applying short π-conjugation length and the donor–acceptor interaction. The high horizontal orientation of the emitter molecule in the film is important to achieve high light out-coupling efficiency. Generally, blue TADF OLED shows the poor lifetime because of the long-delayed exciton lifetime of the emitter. Therefore, short-delayed exciton lifetime is highly required for achieving a long lifetime and high-efficiency OLEDs.

## Several Boron Chemical Structures vs. Their Performances

### Unbridged Boron-Type Acceptor for Blue Thermally Activated Delayed Fluorescence Emitters

**Figure 2:**
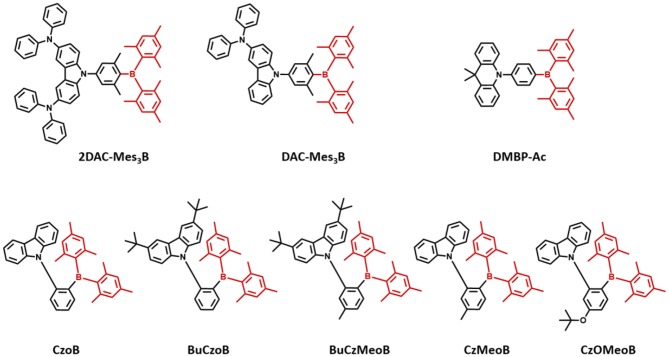
Reported unbridged boron acceptor for blue thermally activated delayed fluorescence (TADF) emitters. In this molecular design, acceptor is highlighted in dark red color.

Triarylboron compounds have been used as optoelectronic materials due to their electron-withdrawing abilities. In 2015, Suzuki et al. (2015) firstly reported a series of triarylboron-based TADF emitters, **2DAC-Mes**_**3**_**B** and **DAC-Mes**_**3**_**B**, by introducing amine-based electron donors. Methyl groups at the *ortho* position of C-B bonds protect the centered boron atom from hydrolysis by oxygen and water. They attached bis(diphenylamino)carbazole (2DAC) and diphenylaminecarbazole (DAC) moieties for electron-donating groups. The **2DAC-Mes3B** and **DAC-Mes3B** showed sky-blue and blue emission peaks at 487 and 477 nm in 16 wt% doped (Bis[2-(diphenylphosphino)phenyl] ether oxide) (DPEPO) film, respectively. These emitters also showed high PLQY of 1.00 and 0.87 and small ΔE_ST_ of 0.058 and 0.062 eV in the doped film state, respectively. The OLED devices showed maximum EQE and the Commission Internationale de l'Éclairage (CIE) color coordinates of 21.6% and (0.18, 0.43) for **2DAC-Mes3B** and 14.0% as well as (0.17, 0.30) for **DAC-Mes3B**. Additionally, Kitamoto et al. (2016) reported another dimesitylarylborane-based blue TADF emitters. They attached carbazole and 9,9-dimethylacridane moieties as electron-donating units (Kitamoto et al., 2016). Both emitters showed blue emission in the toluene solution; however, carbazole-based emitter exhibited a large ΔE_ST_ of 0.456 eV and no TADF characteristics. The ΔE_ST_ and PLQY of **9,9-dimethyl-10-(4-(dimesitylboryl)phenyl)-9,10-dihydroacridine (DMBP-Ac)** were shown to be 0.041 eV and 0.83 in 6 wt% doped 1,3-Bis(N-carbazolyl)benzene (mCP) film. The OLED device exhibited a maximum EQE of 16.0% and the CIE color coordinates of (0.14, 0.24). They successfully demonstrated the promising potential of organoboron compounds as highly efficient blue TADF emitters. In 2017, Lee et al. (2017) reported triarylboron TADF emitters coupled with the donor in *ortho* and *para* positions. *Ortho*-positioned donor–acceptor skeleton possessed small enough ΔE_ST_ to have a good TADF performance, but *para*-positioned emitters had relatively large ΔE_ST_, which was around 0.40 eV. Among *ortho*-positioned emitters, **CzoB** showed a blue emission peak at 466 nm, high PLQY of 0.84, and a small ΔE_ST_ of 0.124 eV in 20 wt% of doped DPEPO film. The OLED device showed a maximum EQE of 22.6% (or 24.1%) and CIE color coordinates of (0.139, 0.150) or (0.139, 0.198) depending on the thickness of indium tin oxide (ITO). They showed that spatially hindered *ortho* donor–acceptor skeleton is effective for achieving small ΔE_ST_ and efficient TADF emitter. Later, the same group enhanced the TADF device performance and emission color by manipulating the electronic structure (Lee et al., 2018). They synthesized a series of donor–acceptor-type emitters based on **CzoB (BuCzoB**, **BuCzMeoB**, **CzMeoB**, and **CzOMeoB)** by introducing substituents into the donor and/or acceptor. They inserted tert-Bu, Me, and OMe into carbazole donor and/or to the phenyl ring of the acceptor. These four emitters showed maximum blue emission peak at 485, 478, 456, and 445 nm in 10–20 wt% doped DPEPO film, respectively. The corresponding PLQYs were 0.91, 0.93, 0.83, and 0.63 and small ΔE_ST_ values from 0.084 to 0.14 eV, respectively. The fabricated TADF devices exhibited electroluminescence peak from deep blue to sky blue and high EQE. **BuCzoB** showed a maximum EQE of 26.1% and CIE of (0.142, 0.344). Also, the **BuCzMeoB** device showed the highest EQE of 32.8% and CIE of (0.135, 0.266). On the other hand, **CzMeoB-** and **CzOMeoB**-based devices showed a maximum EQE of 18.4 and 17.3% and deep blue color coordinates of (0.138, 0.140) and (0.150, 0.086), respectively. This study showed that the *ortho*-carbazole triarylboron compounds can satisfy the high efficiency of blue TADF emitters. Also, by using substituents to donor and/or acceptor position of CzoB backbone molecule, color and efficiency can be adjusted successfully. Triarylboron-based blue TADF emitters are listed in Figure 2 and Table 1.

**Table 1:** Summary of photophysical properties and device performance of the unbridged boron-type acceptor for blue thermally activated delayed fluorescence (TADF) emitters.

**Emitter**	**λ_max_** **[nm]**	**PLQY**	**${\Delta \text{E}}_{\text{ST}}^{\text{d}}$** **[eV]**	**τ_d_** **μs**	**Host**	**EQE_**max**_ [%]**	**CE_**max**_ [cdA^**−1**^]**	**CIE 1931** **(x, y)**	**References**
2DAC-Mes3B	495^a^	1.00	0.058	–	DPEPO (16 wt%)	21.6	–	(0.18, 0.43)	Suzuki et al., 2015
DAC-Mes3B	477^a^	0.87	0.062	–	DPEPO (16 wt%)	14.0	–	(0.17, 0.30)	Suzuki et al., 2015
DMBP-Ac	–	0.83	0.041	6.71^a^	mCP (6 wt%)	16.0	–	(0.14, 0.24)	Kitamoto et al., 2016
CzoB	466^c^	0.84	0.124	56.3 DPEPO (20 wt%)	DPEPO (20 wt%)	22.6	28.5	(0.139, 0.150)	Lee et al., 2017
BuCzoB	485^b^	0.91	0.084	23.0 DPEPO (10 wt%)	DPEPO (10 wt%)	26.1	56.9	(0.142, 0.344)	Lee et al., 2018
BuCzMeoB	478^c^	0.93	0.092	26.6 DPEPO (20 wt%)	DPEPO (20 wt%)	32.8	56.8	(0.135, 0.266)	Lee et al., 2018
CzMeoB	456^c^	0.83	0.13	76.3 DPEPO (20 wt%)	DPEPO (20 wt%)	18.4	20.4	(0.138, 0.140)	Lee et al., 2018
CzOMeoB	445^c^	0.63	0.14	87.3 DPEPO (20 wt%)	DPEPO (20 wt%)	17.3	13.1	(0.150, 0.086)	Lee et al., 2018

a Measured in oxygen-free toluene solution (10^−5^ M).

b Measured in 10 wt% doped film in DPEPO host.

c Measured in 20 wt% doped film in DPEPO host.

*^d^ΔE_ST_ = S_1_-T_1_*.

### Partially Bridged Boron-Type Acceptor for Blue Thermally Activated Delayed Fluorescence Emitters

**Figure 3:**
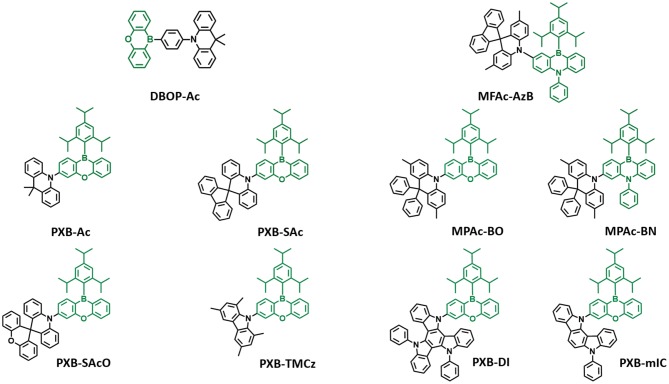
Reported partially bridged boron acceptor for blue thermally activated delayed fluorescence (TADF) emitters. In these molecules, acceptor is highlighted in dark green color.

In 2015, Kitamoto et al. (2015) designed boron-incorporated aromatic moiety into the π-conjugated system. Unlike prior triarylboron compounds, they inserted the boron atom into the closed ring system and connected the phenyl linker and donor in the *para* position. They reported 10H-phenoxaborin acceptor-based blue TADF emitters containing carbazole and 9,9-dimethylacridine donors (Kitamoto et al., 2015). They connected 10H-phenoxaborin acceptor and these donors through a phenyl linker at the 1,4 positions. Therefore, quasi anthracene and fluorine units would have a large dihedral angle between hydrogen atoms of phenyl linker, donor, and boron acceptor, resulting in a small overlap of HOMO and LUMO and small ΔE_ST_ value. Both molecules showed blue emission in the toluene solution. Even though carbazole-based emitter exhibited deep blue emission, due to the large ΔE_ST_ of 0.35 eV, it had no delayed fluorescence component. However, **9,9-dimethyl-10-(4-(10H-phenoxaboryl)phenyl)-9,10-dihydroacridine (DBOP-Ac)** had a small ΔE_ST_ of 0.013 eV and high PLQY of 0.98 in 6 wt% doped DPEPO film. The device showed maximum EQE of 15.1% and EL spectrum peak at 466 nm, indicating that the boron-based acceptor can be promising candidates for developing blue TADF emitter. Meanwhile, Numata et al. (2015) reported four types of blue TADF emitters based on 10H-phenoxaborin acceptor and acridan donor units. Using the electron-withdrawing property of boron atom, bulky 2,4,6-triisoprophylphenyl (TIPP) unit was directly substituted to boron atom so that it can protect the boron atom against the nucleophilic species such as oxygen and water. In addition, acridan donors and 1,3,6,8-tetramethyl-carbazole were selected as the donor moieties due to their good electron-donating property and expectation of large dihedral angle between acceptor arising from the hydrogen atom. Therefore, they synthesized **9,9-dimethyl-10-(10-(2,4,6-triisopropylphenyl)-10H-dibenzo[b,e][1,4]oxaborinin-3-yl)-9,10-dihydroacridine (PXB-Ac)**, **10-(10-(2,4,6-triisopropylphenyl)-10H-dibenzo[b,e][1,4]oxaborinin-3-yl)-10Hspiro[acridine-9,9'-fluorene] (PXB-SAc)**, **10-(10-(2,4,6-triisopropylphenyl)-10H-dibenzo[b,e][1,4]oxaborinin-3-yl)-10Hspiro[acridine-9,9'-xanthene] (PXB-SAcO)**, and **1,3,6,8-tetramethyl-9-(10-(2,4,6-triisopropylphenyl)-10Hdibenzo[b,e][1,4]oxaborinin-3-yl)-9H-carbazole (PXB-TMCz)**. As expected, all emitters exhibited a small ΔE_ST_ of around 0.06–0.12 eV, confirming TADF characteristics. All emitters showed blue emission in toluene solution and maximum PL peaks at 475, 456, 451, and 443 nm and high PLQY of 1.00, 0.76, 0.56, and 0.86 for emitters 1–4, respectively, in 20 wt% doped 2,8-bis(diphenylphosphineoxide)dibenzofuran (PPF) film. The OLED devices based on the TADF emitters (**PXB-Ac, PXB-Sac, PXB-SAcO, and PXB-TMCz)** exhibited high EQE of 21.7, 19.0, 20.1, and 13.3%, respectively, and blue emission. Especially, CIE color coordinates of **PXB-SAcO** and **PXB-TMCz** were (0.14, 0.16). Later, the same group reported phenazineborin-based blue TADF emitter by using spiro-acridan donor, **MFAc-AzB** (Park et al., 2016). For blue emission wavelength, they inserted a phenyl-substituted amino unit in phenazaborin acceptor to weaken the electron-withdrawing ability of the boron atom. Therefore, their synthesized **MFAc-AzB** material showed a blue emission peak at 467 nm in 20 wt% doped PPF film. Moreover, **MFAc-AzB** exhibited a small ΔE_ST_ value of 0.24 eV and PLQY value of 0.99 in 20 wt% doped PPF film and even 0.53 in the neat film state. The **MFAc-AzB** device showed a maximum EQE of 18.2% and CIE coordinates of (0.15, 0.23). Both studies demonstrated the suitability of 10H-phenoxaborin or phenazineborin compounds for highly efficient blue emission TADF materials. Later, Park et al. (2018) studied a series of dibenzoheteraborin-based blue TADF emitters. They designed a boron center and adjustable bridging heteroatoms, such as sulfur, oxygen, or nitrogen and connected to the dimethyl-diphenylacridan (MPAc) donor. Among them, oxygen-containing **MPAc-Bo** and nitrogen-containing **MPAc-BN** emitters showed blue emission in devices. They exhibited emission peak at 483 and 465 nm and a small ΔE_ST_ of 0.024 and 0.05 eV in 50 wt% doped PPF film for MPAc-Bo and MPAc-BN. Moreover, these emitters showed high PLQY values of 0.99 and 0.75 in the same film condition. In the device, **MPAc-Bo** and **MPAc-BN** exhibited sky-blue emission and corresponding CIE color coordinates of (0.16, 0.38) and (0.14, 0.23). The **MPAc-Bo**- and **MPAc-BN-**based devices exhibited maximum EQE of 24.9 and 16.0%, respectively. They developed highly efficient blue TADF emitters utilizing dibenzoheteraborin acceptors such as phenoxaborin and phenazaborin and donor diphenylacridan (MPAc). In 2019, Ahn et al. (2019b) reported two highly efficient blue TADF emitters by using the 10H-phenoxaborin acceptor and two types of indolocarbazole derivative donors, **PXB-mIC** and **PXB-DI**. Due to the weak electron-donating ability of simple carbazole, they introduced carbazole derivative donors such as indolocarbazole and diindolocarbazole (DI). The large electron planes of donors delivered large spatial HOMO volume, leading to high oscillator strength and high PLQY. Also, *meta*-positioned indole moiety caused high steric hindrance between the acceptor and donor, which results in small ΔE_ST_ and good TADF performance. The **PXB-DI** and **PXB-mIC** showed blue emission in the toluene solution (emission peak at 458 and 438 nm) and a small ΔE_ST_ of 0.09 and 0.19 eV, respectively. Moreover, **PXB-DI** showed a high PLQY of about 0.971 in 20% doped bis(diphenylphosphine oxide)dibenzofuran (DBFPO) film. On the other hand, **PXB-mIC** showed PLQY of 0.631 in the same conditions. Interestingly, **PXB-DI** device exhibited a maximum EQE of 37.4% and CIE color coordinates of (0.16, 0.34). Also, **PXB-mIC** device exhibited a maximum EQE of 18.8% and CIE color coordinates of (0.14, 0.18). Especially, **PXB-mIC** exhibited national television system committee (NTSC) blue characteristic of (0.14, 0.08) and 12.5% of maximum EQE in 5-(5-(2,4,6-triiso-propylphenyl)pyridin-2-yl)-5H-benzo[d]benzo[4,5]imidazo[1,2-a]imidazole (PPBI) host (Figure 3).

Later, Agou et al. (2020) reported the pentacyclic ladder-heteraborin blue TADF emitters. Many researchers have investigated the boron-containing π-electron system because of its fascinating properties in terms of TADF emitter. Agou et al. (2006, 2007a,b) reported π-extended ladder-type heteraborins, and they showed novel π-extended ladder-type blue TADF emitters, **MCz-BOBO** and **MCz-BSBS**, by using oxaborin and thiaborin acceptor and MCz donor units, respectively. The **MCz-BOBO** showed a PL emission peak at 476 nm in 20 wt% doped PPF film and a small ΔE_ST_ of 0.01 eV. However, **MCz-BSBS** showed a slightly red-shifted emission peak at 483 nm and ΔE_ST_ of 0.17 eV. Both emitters exhibited high PLQY values of 1.0 and 0.93, respectively. The **MCz-BOBO** device exhibited maximum EQE of 20.1% and CIE color coordinates of (0.13, 0.20). Also, **MCz-BSBS** showed a maximum EQE of 25.9% and CIE color coordinates of (0.14, 0.33). Both π-extended heteraborins and multi heteroatoms-based materials successively demonstrated excellent blue TADF performance and suggested the development of highly efficient blue TADF emitters.

**Figure 4:**
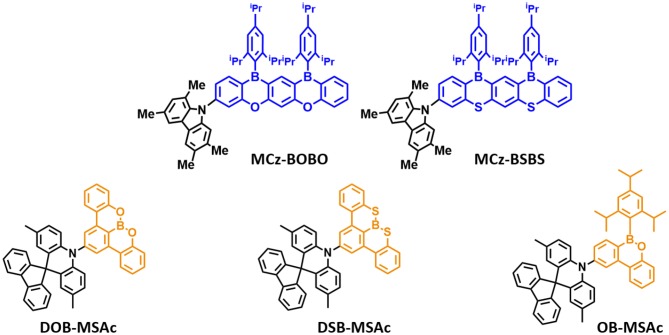
Reported partially bridged boron acceptor for blue thermally activated delayed fluorescence (TADF) emitters. In these molecules, the acceptor is highlighted in blue and yellow color.

In 2019, Matsuo and Yasuda (2019) reported boronate- and borinate-based blue TADF emitters. To accomplish a wider energy gap for blue emission wavelength, they examined boronated and borinated esters. They expected that nearby oxygen atoms can donate electrons and diminish the electron deficiency of the boron atom and decrease the electron-withdrawing ability of boron acceptors. The superiority of borinate- and boronate-containing TADF emitters has not been elucidated yet. Therefore, they synthesized two types of boronate TADF emitter, **10-(8,9-dioxa-8a-borabenzo[fg]tetracen-2-yl)-2,7-dimethyl-10H-spiro[acridine-9,9'-fluorene] (DOB-MSAc)** and **10-(8,9-dithia-8a-borabenzo[fg]tetracen-2-yl)-2,7-dimethyl-10H-spiro[acridine-9,9'-fluorene]** (**DSB-MSAc)**, and one borinate-based TADF emitter, **2,7-dimethyl-10-(6-(2,4,6-triisopropylphenyl)-6H-dibenzo[c,e][1,2]oxaborinin-9-yl)-10H-spiro [acridine-9,9'-fluorene]** (**OB-MSAc)**, using spiro[2,7-dimethylacridan-9,9'-fluorene] as a donor moiety. Boronate-based TADF emitters **DOB-MSAc** and **OB-MSAc** showed PL peaks at 462 and 448 nm in toluene solution, which was significantly blue-shifted compared to that of phenoxaborin-based TADF emitter **MPAc-BO** (477 nm). However, boronate–thioester-based emitter (**DSB-MSAc)** showed a PL peak at 483 nm, and this was slightly blue-shifted compared to the **MPAc-BS** (493 nm). These results are attributed to the decreased electron-accepting ability by replacing of the B-C bond with B-O or B-S bond. Their PLQY and PL peak values in 20 wt% doped PPF films were 0.28, 0.81, and 0.53, and 470, 491, and 458 nm, respectively. They also had a small ΔE_ST_ of 0.30, 0.12, and 0.06 eV for **DOB-MSAc, DSB-MSAc**, and **OB-MSAc**, respectively. In devices, **DOB-MSAc** and **OB-MSAc** exhibited the maximum EQE (CIE color coordinates) of 5.2% (0.16, 0.22) and 12.8% (0.15, 0.15), respectively. Especially, **DSB-MSAc** showed the highest maximum EQE of 20.9% and CIE color coordinates of (0.17, 0.39) due to its high PLQY. They demonstrated a new molecular design for blue TADF by changing heteroatoms directly connected to the boron atom. The abovementioned partially bridged boron blue TADF emitters are listed in Figure 4 and Table 2.

**Table 2:** Summary of photophysical properties and device performance of the partially bridged boron-based type blue thermally activated delayed fluorescence (TADF) emitters.

**Emitter**	**λ_max_** **[nm]**	**PLQY**	**${\Delta \text{E}}_{\text{ST}}^{\text{d}}$** **[eV]**	**τ_d_** **μs**	**Host**	**EQE_**max**_ [%]**	**CE_**max**_ [cdA^**−1**^]**	**CIE 1931** **(x, y)**	**References**
DBOP-Ac	–	0.98	0.013	–	DPEPO (6 wt%)	15.1	–	–	Kitamoto et al., 2015
PXB-Ac	475^a^	1.00	0.10	1.60^a^	PPF (50 wt%)	21.7	–	–	Numata et al., 2015
PXB-Sac	456^a^	0.76	0.12	4.02^a^	PPF (20 wt%)	19.0	–	–	Numata et al., 2015
PXB-SAcO	451^a^	0.56	0.06	2.06^a^	PPF (20 wt%)	20.1	–	(0.14, 0.16)	Numata et al., 2015
PXB-TMCz	443^a^	0.86	0.12	3.49^a^	PPF (20 wt%)	13.3	–	(0.14, 0.16)	Numata et al., 2015
MFAc-AzB	467^c^	0.99	0.24	91 PPF (20 wt%)	PPF (20 wt%)	18.2	32.6	(0.15, 0.23)	Park et al., 2016
MPAc-BO	483^b^	0.99	0.024	1.8 PPF(50 wt%)	PPF (50 wt%)	24.9	57.0	(0.16, 0.38)	Park et al., 2018
MPAc-BN	465^b^	0.75	0.05	18 PPF (50 wt%)	PPF (50 wt%)	16.0	23.7	(0.14, 0.23)	Park et al., 2018
PXB-mIC	438^a^	0.631	0.19	3.89/4.04 DBFPO/PPBI (20 wt%)	DBFPO/PPBI (20 wt%)	18.8/12.5	22.1/8.6	(0.14, 0.18)/ (0.14, 0.08)	Ahn et al., 2019b
PXB-DI	458^a^	0.971	0.09	2.57/3.33 DBFPO/PPBI (20 wt%)	DBFPO/PPBI (20 wt%)	37.4/28.4	66.2/37.8	(0.16, 0.34)/ (0.14, 0.20)	Ahn et al., 2019b
MCz-BOBO	476^c^	1.00	0.01	0.78 PPF (20 wt%)	PPF (20 wt%)	20.1	31.5	(0.13, 0.20)	Agou et al., 2020
MCz-BSBS	483^c^	0.93	0.17	2.7 PPF (20 wt%)	PPF (20 wt%)	25.9	52.9	(0.14, 0.33)	Agou et al., 2020
DOB-MSAc	470^c^	0.28	0.30	140 PPF (20 wt%)	PPF (20 wt%)	5.2	–	(0.16, 0.22)	Matsuo and Yasuda, 2019
DSB-MSAc	491^c^	0.81	0.12	22 PPF (20 wt%)	PPF (20 wt%)	20.9	–	(0.17, 0.39)	Matsuo and Yasuda, 2019
OB-MSAc	458^c^	0.53	0.06	12 PPF (20 wt%)	PPF (20 wt%)	12.8	–	(0.15, 0.15)	Matsuo and Yasuda, 2019

a Measured in oxygen-free toluene solution (10^−5^ M).

b Measured in 50 wt% doped film in PPF host.

c Measured in 20 wt% doped film in PPF host.

*^d^ΔE_ST_ = S_1_-T_1_*.

**Figure 5:**
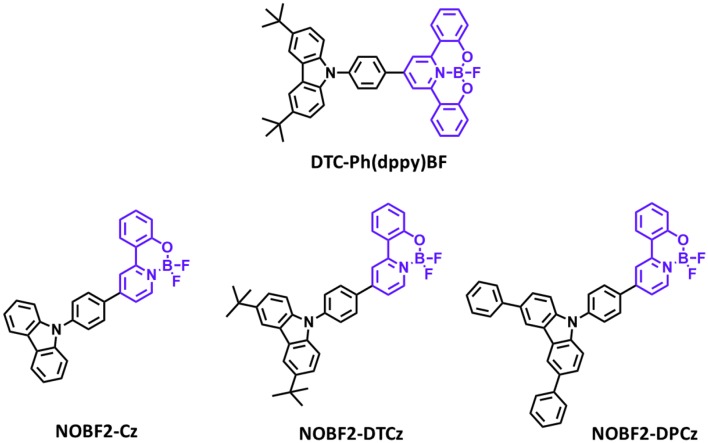
Reported partially bridged boron acceptors for blue thermally activated delayed fluorescence (TADF) emitters. In these molecules, acceptor is highlighted in purple color.

Later, Li P. et al. (2019) reported a new class of four-coordinate fluoroboron acceptor TADF emitters for the first time. They supposed that susceptible cleavage of B-C bond and vacant p-orbital on central boron atom can be the critical reason for short device lifetime. Therefore, they designed four-coordinate boron-centered tridentate 2,2′-(pyridine-2,6-diyl) diphenolate (dppy) ligand, which is O∧N∧O type of chelate anticipating better chemical and thermal stabilities. They employed 3,6-di-tert-butylcarbazole (DTC) as an electron donor moiety. **DTC-Ph(dppy)BF** showed the blue emission peak at 468 nm and a small ΔE_ST_ of 0.27 eV in the toluene solution. Also, the PLQY value was 0.71 in 5 wt% doped of mCP film. Due to the relatively large ΔE_ST_ value, RISC efficiency was limited, and maximum EQE was up to 8.8% and CIE color coordinates of (0.16, 0.31). However, the half-lifetime at a constant driving current density of 10 mA/cm^2^ reached to 2,354 h. They demonstrated the first trial of design and synthesis of four-coordinate boron and tridentate dppy ligand-based blue TADF emitters. Later, Li G. et al. (2019) reported a new series of tetracoordinated blue TADF emitters using donor–acceptor BF2-type skeleton. Tetracoordinated difluoroboron molecules are air-stable, and they can enhance the molecular rigidity to have a high PLQY. They synthesized a series of BF2-type acceptor using different donors, especially **NOBF2-Cz**, **NOBF2-DTCz**, **NOBF2-DPCz**, and **NOBF2-DMAC**. Except for **NOBF2-DMAC**, the other three emitters showed blue emission in the toluene solution from 449 to 473 nm and a small ΔE_ST_ of around 0.20 eV. The PLQY values of **NOBF2-Cz**, **NOBF2-DTCz**, and **NOBF2-DPCz** in 10 wt% doped DPEPO film were 0.99, 0.74, 0.70, respectively. In device, they showed the maximum EQE and CIE color coordinates of 11.0% and (0.14, 0.16) for **NOBF2-Cz**, 12.7% and (0.14, 0.21) for **NOBF2-DTCz**, and 15.8% and (0.14, 0.28) for **NOBF2-DPCz**. They demonstrated that tetracoordinated difluoroboron-based materials can act as stable and efficient TADF emitters (Figure 5 and Table 3).

**Table 3:** Summary of photophysical properties and device performance of the tetracoordinated difluoroboron-based blue thermally activated fluorescence (TADF) emitters.

**Emitter**	**λ_max_** **[nm]**	**PLQY**	**ΔE_**ST**_^**b**^ [eV]**	**τ_d_μs**	**Host**	**EQE_**max**_ [%]**	**CE_**max**_ [cdA^**−1**^]**	**CIE 1931** **(x, y)**	**References**
DTC-Ph(dppy)BF	468^a^	0.71	0.27	–	mCBP (14 wt%)	8.8	17.9	(0.16, 0.31)	Li P. et al., 2019
NOBF2-Cz	449^a^	0.99	0.20	132 DPEPO (10 wt%)	mCBP (10 wt%)	11.0	12.6	(0.14, 0.16)	Li G. et al., 2019
NOBF2-DTCz	473^a^	0.74	0.20	126 DPEPO (10 wt%)	mCBP (10 wt%)	12.7	40.6	(0.14, 0.21)	Li G. et al., 2019
NOBF2-DPCz	471^a^	0.70	0.22	110 DPEPO (10 wt%)	mCBP (10 wt%)	15.8	25.3	(0.14, 0.28)	Li G. et al., 2019

a Measured in oxygen-free toluene solution (10^−5^ M).

b *ΔE_ST_ = S_1_-T_1_*.

Three-coordinate organoborane compounds successfully act as electron acceptors in the blue TADF, and in most cases, reactive boron center is surrounded by sterically bulky 2,4,6-trimethylphenyl (Mes) groups. Although these bulky aryl groups protect the center boron from hydrolysis and unstable conditions, highly steric moieties disturb intermolecular interaction and cause detrimental results in solid-state performance. And the sole boron atom itself was not enough to satisfy the deep blue emission. Therefore, researchers searched the alternative strategy not only stabilizes the boron but also tunes deeper blue emission, so that they inserted the boron atom in π-conjugated system with heteroatoms. This strategy enables us to have a structurally constraint skeleton, good molecular rigidity, and intermolecular interaction in solid state. Secondly, by introducing the heteroatom, better TADF properties and pure blue color were achieved, demonstrating partially bridged boron-based TADF more sufficiently satisfied the requirement of blue material.

### Oxygen-Based Fully Bridged Boron-Type Acceptor for Blue Thermally Activated Delayed Fluorescence Emitters

Recently, triarylboron-based acceptors consisting bridged cyclized unit in donor–acceptor structure have reported good blue TADF performance. Normally, both nitrogen- or oxygen-based fully bridged boron-type acceptor-based materials showed deep-blue emission, narrow FWHM, and good TADF performance. Therefore, such acceptor structure can be used for the design of efficient and stable TADF emitters.

Recently, very few oxygen-based fully bridged boron type TADF emitters have reported by attaching a donor unit to the central phenyl ring of the oxygen-based fully bridged boron unit on the para-position of the boron atom. The initial sky-blue TADF emitter containing oxygen-based fully bridged boron acceptor and phenoxazine donor (**10-(5,9-dioxa-13b-branaphtho[3,2,1-de]anthracene-7-yl)-10H-phenoxazine (DOBNA-PX**) was reported by Hirai et al. (2015) in 2015. Their emitter showed a sky-blue emission (492 nm), absolute PLQY of 0.92 in 1 wt% doped poly(methyl methacrylate) (PMMA) film, and a small ΔE_ST_ of 0.06 eV due to the localization of the single occupied molecular orbitals (SOMOs). They showed an EQE of about 13.9% for the **DOBNA-PX-**based OLED. Though the performances of **DOBNA-PX** emitters are lower than the expectation, this study showed the suitable properties of blue TADF emitters. This acceptor moiety can also act as a strong electron acceptor, and it can be suitable to achieve horizontal molecular orientation because of its rigid and symmetrical configuration (Kim and Kim, 2018).

**Figure 6:**
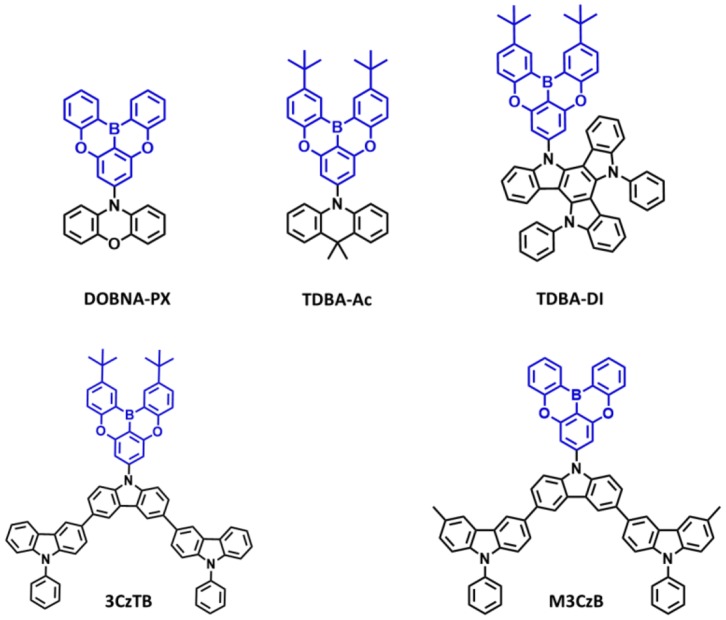
Reported structures of blue thermally activated delayed fluorescence (TADF) emitters using oxygen-based fully bridged boron acceptor and different donors.

Secondly, in 2019, Ahn et al. (2019a) reported two new highly efficient blue TADF emitters, **TDBA–Ac** and **TDBA–DI** (Figure 6). To satisfy the requirements of the blue TADF materials, they used oxygen-based fully bridged triarylboron with tert-butyl group on the periphery of terminal phenyl ring as an acceptor and DI or acridine (Ac) as donor in the emitter structure. They found that rigid ring-based DI can be used to achieve a large twist angle with an acceptor (Kim et al., 2018). Both emitters, **TDBA-Ac** and **TDBA-DI**, showed a deep-blue emission and maximum PL peak at 458 and 456 nm, respectively, in solution state. These emitters also exhibited a narrow FWHM of about 50 (**TDBA-Ac**) and 55 nm (**TDBA-DI**). The calculated singlet and triplet energies from the onset of the RTPL and LTPL spectra of **TDBA-Ac** and **TDBA-DI** were 3.11/3.05 and 3.06/2.95 eV, respectively. Both of these emitters exhibited small ΔE_ST_ values of 0.06 (in solution state) and 0.05 eV (20 wt% doped **TDBA-DI** in DBFPO film) for **TDBA-Ac** and **TDBA-DI**, respectively. They found a delayed exciton lifetime of 1.82 and 1.79 μs for **TDBA-Ac** and **TDBA-DI**, respectively, which was shorter than the reported blue TADF emitters. **TDBA-Ac** (20 wt%) and **TDBA-DI** (20 wt%) in the DBFPO host showed a PLQY of 0.93 and 0.99, respectively. Also, the HOMO dispersing molecular design of emitters and low non-radiative decay rates were responsible for achieving high PLQY. The **TDBA-Ac-** and **TDBA-DI**-based OLEDs (DBFPO host) exhibited maximum EQE of 25.71 and 38.15%, respectively. The **TDBA-DI** device showed a maximum luminance of 47,680 cd/m^2^ and a horizontal dipole orientation ratio of 0.89. Such high efficiency, high luminance, and good color coordinates were attributed to the highly conjugated and rigid donor and acceptor combination for high PLQY and narrow emission, the large dihedral angle between the donor and acceptor for small ΔE_ST_, fast RISC, and horizontal molecular orientation. Also, the **TDBA-DI** emitter and mCBP-CN host system device showed an operational lifetime (T_50_) of 55.2 h at the initial luminance of 1,000 cd/m^2^. They also reported **DBA-DI** emitter by removing tert-butyl from the **TDBA-DI** to achieve high efficiency and long lifetime (Ahn et al., 2020). The **DBA-DI** showed slight variations in the photophysical properties compared to the **TDBA-DI** as shown in Table 4. However, the **DBA-DI** device exhibited high EQE of 26.4%, CIE color coordinates of (0.17, 0.40), and lifetime of 540 h at the initial luminance of 1,000 cd/m^2^.

**Table 4:** Summary of photophysical and device characteristics of the oxygen-based fully bridged boron type blue thermally activated delayed fluorescence (TADF) emitters.

**Emitter**	**λ_max_** **[nm]**	**PLQY**	**${\Delta \text{E}}_{\text{ST}}^{\text{d}}$** **[eV]**	**τ_d_** **μs**	**Host**	**EQE_**max**_ [%]**	**CE_**max**_ [cdA^**−1**^]**	**CIE 1931** **(x, y)**	**References**
DOBNA-PX	492^c^	0.92	0.06	31.8 PMMA (1wt%)	CBP (20 wt%)	13.9	40.1	–	Hirai et al., 2015
TDBA-Ac	458^a^	0.93	0.06	1.82 DBFPO (20 wt%)	DBFPO (20 wt%)	25.71	27.73	(0.14, 0.15)	Ahn et al., 2019a
TDBA-DI	456^a^	0.99	0.05	1.79 DBFPO (20 wt%)	DBFPO (20 wt%)	38.15	64.38	(0.15, 0.28)	Ahn et al., 2019a
DBA-DI	467^a^	95.3	0.03	1.25 mCBP-CN (30 wt%)	mCBP-CN: DDBFT (30 wt%)	26.4	–	(0.17, 0.40)	Ahn et al., 2020
3CzTB	433^a^	0.87	0.23	9.32 DBFPO (20 wt%)	DBFPO (20 wt%)	29.1	36.4	(0.14, 0.19)	Karthik et al., 2020
M3CzB	445^a^	0.92	0.14	7.84 DBFPO (20 wt%)	DBFPO (20 wt%)	30.7	46.7	(0.14, 0.26)	Karthik et al., 2020
OBA-O	444^a^	0.84	0.09	4.14 mCP (10 wt%)	mCP (5 wt%)	17.8	33.2	(0.17, 0.17)	Song et al., 2019
OBA-S	456^a^	0.75	0.09	4.80 mCP (10 wt%)	mCP (5 wt%)	4.4	6.3	(0.20, 0.31)	Song et al., 2019
OBA-BrO	470^a^	0.92	0.04	3.74 mCP (10 wt%)	mCP (5 wt%)	22.5	49.2	(0.21, 0.38)	Song et al., 2019
OBA-BrS	478^a^	0.55	0.07	0.81 mCP (10 wt%)	mCP (5 wt%)	9.2	20.3	(0.29, 0.46)	Song et al., 2019
m-AC-DBNA	492^b^	0.89	0.009	7.6 BCPO (5 wt%)	BCPO (10 wt%)	17.1	42.0	(0.18, 0.42)	Meng et al., 2019
m'-AC-DBNA	498^b^	0.96	0.031	1.5 BCPO (5 wt%)	BCPO (10 wt%)	14.1	35.3	(0.18, 0.39)	Meng et al., 2019
p-AC-DBNA	496^b^	0.87	0.009	7.8 BCPO (5 wt%)	BCPO (10 wt%)	20.5	47.4	(0.17, 0.36)	Meng et al., 2019

a Measured in toluene solvent.

b Measured in 10 wt% doped film in BCPO host.

c Measured in 1 wt% doped film in PMMA host.

*^d^ΔE_ST_ = S_1_-T_1_*.

Later, they reported a series of blue TADF emitters consisting of oxygen-based fully bridged triarylboron acceptor and tercarbazole donor. These two emitters, **3CzTB** and **M3CzB**, were designed by alkyl modification on the donor and acceptor units, which changes not only their donor–acceptor interaction but also the electronic nature (Karthik et al., 2020). 6,6′-Dimethyl substituted tercarbazole moiety was used as a donor unit and attached to the oxygen-based fully bridged triarylboron acceptor (**M3CzB**) to improve the electrochemical stability of the TADF emitter. The tert-butyl-modified boron acceptor has attached to the methyl group-modified tercarbazole donor (**3CzTB**) to tune the acceptor strength. These emitters, **3CzTB** and **M3CzB**, showed emission wavelength and ΔE_ST_ of 433 nm and 0.23 eV and 445 nm and 0.14 eV, respectively, in the solution state. Also, **3CzTB** and **M3CzB** showed a delayed exciton lifetime of 9.32 and 7.84 μs, respectively, which was measured from the 20 wt% doped DBFPO films. However, **M3CzB** showed a higher RISC rate and lower triplet non-radiative rate compared to the **3CzTB** because of its low ΔE_ST_. The **3CzTB-** and **M3CzB-**based OLEDs exhibited a high EQE of 29.1 and 30.7%, respectively, which was attributed to the high PLQY, fast RISC, and horizontal dipole orientation of the emitter. These devices also showed a maximum luminance of 18,160 cd/m^2^ for **M3CzB** and 11,690 cd/m^2^ for **3CzTB**.

**Figure 7:**
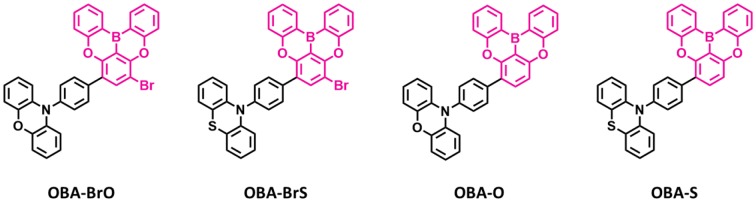
Reported structures of blue thermally activated delayed fluorescence (TADF) emitters using oxygen-based fully bridged boron acceptor and different donors.

Recently, several other TADF materials were synthesized by attaching different donor units to the central phenyl ring of the oxygen-based fully bridged boron acceptor (**OBA**) on the meta-position of the boron. Song et al. (2019) reported four new TADF emitters, **OBA-O**, **OBA-S**, **OBA-BrO**, and **OBA-BrS**, by using oxygen-based fully bridged boron acceptors (**OBA or DBA**) and phenoxazine or phenothiazine donors (Figure 7). Also, they attached Br- group to the **OBA** unit (**OBA-Br**) to improve the TADF properties of the emitter. The phenothiazyl-based emitters showed longer maximum emission wavelengths of 456 (**OBA-S**) and 478 nm (**OBA-BrS**) in the solution state compared to phenoxazyl-based materials (**OBA-O**: 444 nm and **OBA-BrO**: 470 nm) because of stronger donating ability of the phenothiazyl. The blue TADF emitter **OBA-O** and its blue analog **OBA-BrO** showed higher PLQY values of 0.84 and 0.92, respectively, than both **OBA-S** (0.75) and **OBA-BrS** (0.55). All these emitters showed very small ΔE_ST_ values <0.1 eV and good TADF properties due to the large twist angle near to 90° between the donor and acceptor, which makes separation of the natural transition orbitals. They achieved ΔE_ST_ of 0.09 eV for **OBA-O** and **OBA-S**, while 0.04 and 0.07 eV for **OBA-BrO** and **OBA-BrS**, respectively. On the other hand, both -Br-attached TADF emitters showed shorter delayed exciton lifetime (higher K_RISC_) of 3.74 (8.97 × 10^5^ s^−1^) and 0.81 μs (8.41 × 10^5^ s^−1^) for OBA-BrO and OBA-BrS, respectively, compared to OBA-O (4.14 μs and 4.28 × 10^5^ s^−1^) and OBA-S (4.80 μs and 2.76 × 10^5^ s^−1^) due to their smaller ΔE_ST_ values. Such a high K_RISC_ was attributed to the electron-withdrawing ability of -Br group on the OBA acceptor that will improve the TADF process in **OBA-BrO** and **OBA-BrS**. **OBA-O** and **OBA-BrO** devices showed higher EQE of 17.8 and 22.5% than **OBA-S** (4.4%) and **OBA-BrS** (9.2%) devices because of their higher PLQY values. Among these TADF materials, **OBA-O** device showed the bluest CIE color coordinates (0.17, 0.17) and a maximum EL peak of 446 nm. However, other emitter-based devices showed sky-blue CIE color coordinates (Table 4).

**Figure 8:**
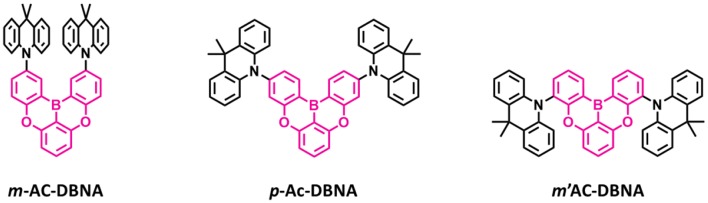
Reported structures of blue thermally activated delayed fluorescence (TADF) emitters using donor units attached to the different positions of the boron-based acceptor.

Also, another series of TADF emitters were reported by using donor unit into the different positions of the oxygen-based fully bridged boron acceptor. Meng et al. (2019) reported the effect of substitutional positions of the donor group on the TADF properties of the oxygen-based fully bridged boron-type acceptor-based emitter. They synthesized three different boron-based TADF emitters, **m-Ac-DBNA**, **p-AC-DBNA**, and **m'-AC-DBNA**, by using two dimethyl Ac units at meta-, para-, and meta'-positions of the 5,9-dioxa-13b-boranaphtho[3,2,1-de]anthracene (DBNA) (Figure 8). They selected Ac as a donor unit because of its strong electron-donating property and suitable steric hindrance for the TADF molecule. They checked an effect of such molecular design on intermolecular interactions, thermal and photophysical properties, and EL performance. All three emitters showed an emission peak at 514–518-nm range (film state). However, PL properties of 5 wt% emitter doped **BCPO** films showed sky-blue emission, which were blue-shifted by about 20 nm than the neat films. A para isomer showed higher PLQY of 0.96 than m- (0.89) and m'- (0.87) isomers in the doped film. The ΔE_ST_ values of all three emitters were measured to be 9.0 (**m-AC-DBNA**), 9.1 (**p-AC-DBNA**), and 31 (**m'-AC-DBNA**) meV. Among all the three isomers, **p-Ac-DBNA** exhibited the highest K_RISC_ value of 1.17 × 10^6^ s^−1^ and delayed exciton lifetime of 1.5 μs. The OLEDs (10% doped TADF materials) exhibited maximum EQE of 17.1, 14.1, and 20.5% for m-, m'-, and p-isomers, respectively. On the other hand, neat p-isomer device showed an excellent maximum EQE of 14.1%. This result shows the doping concentration-independent characteristic of these TADF emitters. The TADF devices (m-, m'-, and p-isomers) exhibited maximum EL peak at 492–488 nm and sky-blue CIE color coordinates.

The above-described TADF emitters containing oxygen-based fully bridged boron acceptors and different donor units exhibited good TADF properties and high efficiency. Interestingly, among the above-described oxygen-based fully bridged boron-type acceptor-based TADF emitters, the design containing donor units attached to the central phenyl ring of the oxygen-based fully bridged boron unit on the para-position of the boron atom exhibited excellent TADF and OLED properties.

### Multiple Resonance-Based Structure for Blue Thermally Activated Delayed Fluorescence Emitters

Recently, multiple resonance-TADF (MR-TADF) materials showed good electroluminance properties due to their high efficiency and color purity. Generally, these materials show very narrow emission profiles because of their rigid and symmetrical structures. Moreover, MR-TADF materials show majorly deep blue emission with high color purity. Unlike the traditional donor–acceptor-type TADF configuration, this type of MR-TADF materials show HOMO and LUMO orbital separation at *ortho* and *para* positions with respect to nitrogen and boron atoms, respectively, within the same conjugated planar core structure. Moreover, this type of materials shows high oscillator strength along with small ΔE_ST_, which is contradictory to conventional TADF materials. Though MR-TADF materials possess several advantages over conventional TADF emitters, they suffer from very limited chemical modification, complicated synthesis, and high-efficiency roll-off. Additionally, the underlying mechanism behind the high oscillator strength along with small ΔE_ST_ is still not clearly known. However, Pershin et al. (2019) studied the insightful mechanism of multiple resonance-based materials by performing Spin-Component Scaling second-order approximate Coupled-Cluster (SCS-CC2) theoretical calculations. The insightful mechanism was predicted by using the wave functions of two metrics such as the electron-hole charge separation distance (Δ*r*) and the amount of charge transferred (Δ*q*) between them at the lowest singlet excited state. From there, it was found that a substantial reshuffling of electron density (large Δ*q*) occurs over short distances (small Δ*r*), i.e., the “short-range/local CT” state features both high electron-hole wave function overlaps and small exchange interactions resulting in high oscillator strength and small ΔE_ST_, respectively. Further, these behaviors increase as proportional to the increase in molecular conjugation.

**Figure 9:**
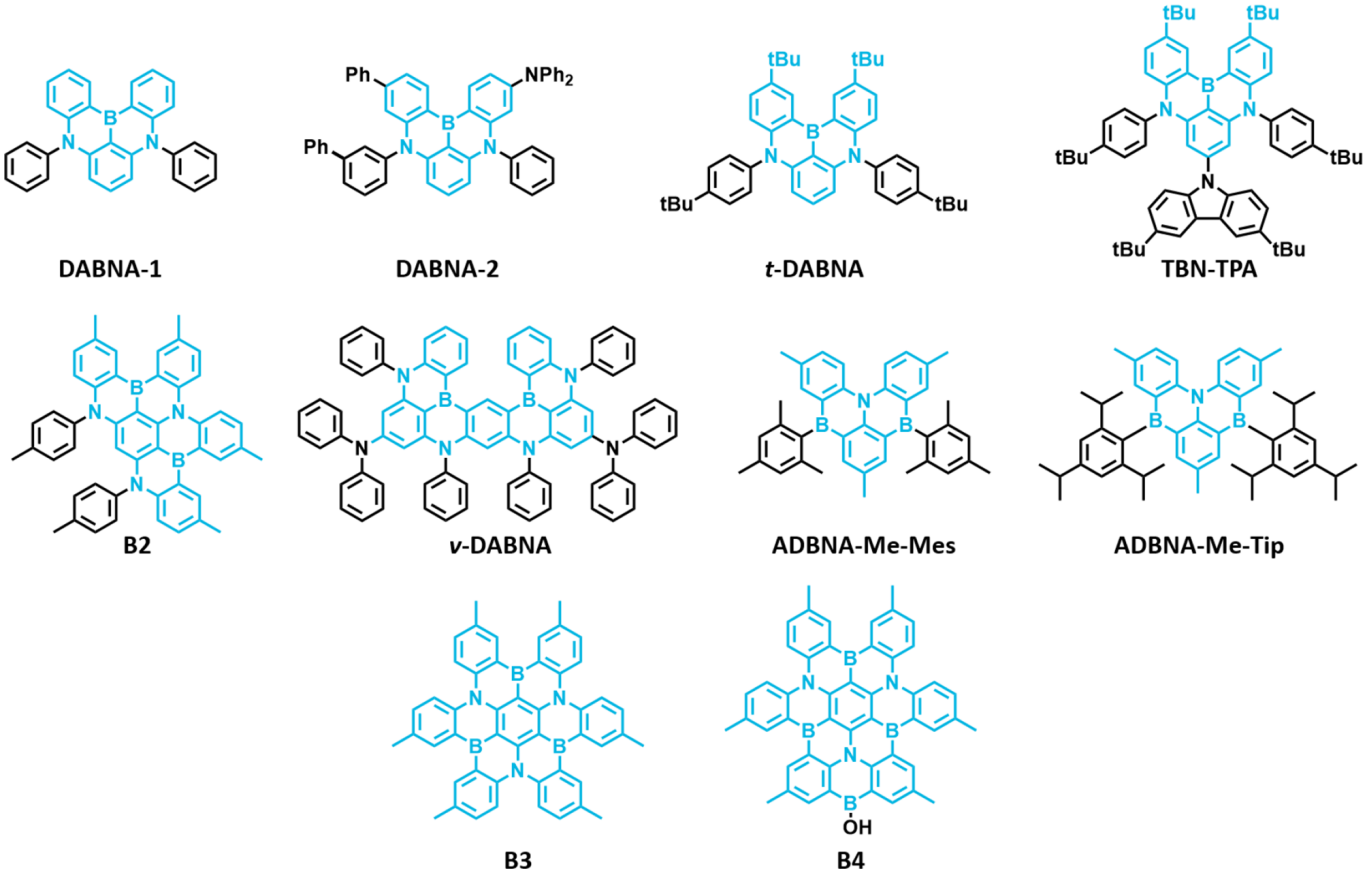
Reported structures of multiple resonance (MR)-thermally activated delayed fluorescence (TADF)-based materials for blue TADF.

The first MR-TADF materials containing one boron atom and two nitrogen atoms, **DABNA-1** and **DABNA-2**, were reported by Hatakeyama et al. (2016) in 2016. These materials exhibited deep blue emission with PLQY and ΔE_ST_ of 0.88 and 0.18 eV and 0.90 and 0.15 eV in 1 wt% doped mCBP film for **DABNA-1** and **DABNA-2**, respectively. Also, they showed high EQE values of 13.5% and 20.2% with deep blue color coordinates of (0.13, 0.09) and (0.12, 0.13) along with very narrow EL spectra of FWHM 28 nm for **DABNA-1** and **DABNA-2**, respectively. The high color purity of these materials is attributed to narrow EL spectra with very small FWHM of 28 nm for both materials. Though these materials showed very high efficiency roll-off, they are promising candidates for developing pure deep blue TADF materials. Later, Han et al. (2019) reported the *tert*-butyl-modified **DABNA-1**, ***t*-DABNA**, which was used with TADF assistant dopant **DMAC-DPS** to increase the device efficiency and to reduce roll-off. This assistant dopant device exhibited improved maximum efficiency from 25.1 to 31.4% and reduced roll-off compared to without assistant dopant device. In another report, ***t*-DABNA** structure was modified with the addition of carbazole donor on the *para* position to the boron atom to get **TBN-TBA** (Liang et al., 2018). The addition of donor segment gave high PLQY of up to 0.975 and very small ΔE_ST_ of 0.03 eV in toluene solution. Also, the blue TADF OLED fabricated with 4 wt% **TBN-TBA** in **2,6-DCzppy** host showed very high EQE of 32.1% with EL maximum of 447 nm and FWHM of 27 nm. Matsui et al. (2018) reported di- and tri-boron doped nanographenes **B2**, **B3**, and **B4** as multiple resonance TADF materials. All materials showed deep blue emission in the range from 441 to 455 nm with small FWHM of 32–34 nm in 1 wt% doped in PMMA films (Matsui et al., 2018). The PLQY values are in the range of 0.33−0.57 and small ΔE_ST_ values in the range of 0.15 eV−0.18 eV in PMMA films. The TADF device with **B2** as 1 wt% emitting dopant in mCBP host exhibited maximum EQE of 18.3% with EL peak maximum of 460 nm and FWHM of 37 nm. Later, Kondo et al. (2019) reported a dimer of **DABNA-1** core, **ν-DABNA** with two boron atoms and two diphenylamine donors attached para positions to the boron atoms. The photophysical properties of the **ν-DABNA** in 1 wt% doped in **DOBNA-OAr** host showed a deep blue emission peak, PLQY, and ΔE_ST_ of 467 nm, 0.9, and 17 meV, respectively. Though the conjugation of **ν-DABNA** increased doubly compared to **DABNA-1**, the emission peak is retained in the blue region as in **DABNA-1**. But the increase in PLQY and reduction in ΔE_ST_ values are observed for **ν-DABNA** compared to **DABNA-1**. The TADF device based on **ν-DABNA** showed the highest EQE of 34.4% with deep blue CIE coordinates of (0.12, 0.11) with very narrow FWHM of 18 nm. Also, this device showed very low efficiency roll-off of only 8.6% at 1,000 cd/m^2^. This is the highest efficiency of multiple resonance-based TADF materials reported so far. Recently, Nguyen et al. (2020) evaluated the performances of exciplex host-based TADF devices, and they compared the device performances with and without **ν-DABNA** as 1 wt% emitting dopant. The maximum EQE and brightness of 8.9% and 830 cd/m^2^ and 19% and 1,260 cd/m^2^ for the devices with and without **ν-DABNA** were observed. Further, the LT_50_ lifetime of both devices showed almost similar values (~350 h) and indicates the promising way of making stable devices (Nguyen et al., 2020). Recently, Oda et al. (2019) reported two multiple resonance-based TADF materials, **ADBNA-Me-Mes** and **ADBNA-Me-Tip**; these materials possess one nitrogen atom in the center of the core and two boron atoms in the outer of the core unit. It is interesting to note that on incorporation of two electron withdrawing boron atoms in the **ADBNA** core, the emission peak is red-shifted about 20 nm compared to **DABNA-1**. Though these two materials showed similar PLQY values compared to **DABNA-1**, they showed little higher ΔE_ST_ values than **DABNA-1**. The TADF OLEDs using **ADBNA-Me-Mes** and **ADBNA-Me-Tip** as emitting dopant exhibited sky-blue emission with maximum EQE of 21.4 and 16.2% with little broad emission spectrum (FWHM of 32 and 33 nm), respectively. Apart from blue emission, green multiple resonance-based TADF materials were reported by Zhang et al. (2019) by incorporating weak acceptor groups on the periphery of the DABNA core. These results indicate that incorporation of a donor group at the *para* position to boron atom on the DABNA core retains the deep blue color, whereas the acceptor group incorporation gives a red-shifted emission color (Figure 9 and Table 5).

**Table 5:** Summary of photophysical properties and device performances of the multiple resonance (MR)-thermally activated delayed fluorescence (TADF)-based blue TADF materials.

**Emitter**	**λ_max_** **[nm]**	**PLQY**	**${\Delta \text{E}}_{\text{ST}}^{\text{e}}$** **[eV]**	**τ_d_** **μs**	**Host**	**EQE_**max**_ [%]**	**CE**_**max**_ **[cdA**^**−1**^**]**	**CIE 1931** **(x, y)**	**References**
DABNA-1	460^a^	0.88	0.18	93.7 mCBP (1 wt%)	mCBP (1 wt%)	13.5	10.6	(0.13, 0.09)	Hatakeyama et al., 2016
DABNA-2	469^a^	0.90	0.14	65.3 mCBP (1 wt%)	mCBP (1 wt%)	20.2	21.1	(0.12, 0.13)	Hatakeyama et al., 2016
B2	455^b^	0.53	–	30.4 PMMA (1 wt%)	mCBP (1 wt%)	18.3	16.7	(0.13, 0.11)	Matsui et al., 2018
B3	441^b^	0.33	–	–	–	–	–	–	Matsui et al., 2018
B4	450^b^	0.57	–	–	–	–	–	–	Matsui et al., 2018
TBN-TPA	470^c^	0.97	0.14	51.02 DCzppy (4 wt%)	2,6-DCzppy (4 wt%)	32.1	40.2	(0.12, 0.19)	Liang et al., 2018
*t*-DABNA	–	0.85	0.17	83.3 DPEPO (-wt%)	DPEPO (5 wt%)	25.1	13.0	–	Han et al., 2019
ν-DABNA	467^d^	0.90	0.017	4.1 DOBNA-Oar (1 wt%)	DOBNA-Oar (1 wt%)	34.4	31.0	(0.12, 0.11)	Kondo et al., 2019
ADBNA-Me-Mes	482^d^	0.89	0.20	165 DOBNA-Oar (1 wt%)	DOBNA-Oar (1 wt%)	16.2	15.4^f^	(0.10, 0.27)	Oda et al., 2019
ADBNA-Me-Tip	479^d^	0.88	0.19	147 DOBNA-OAr (1 wt%)	DOBNA-Oar (1 wt%)	21.4	23.5^f^	(0.11, 0.29)	Oda et al., 2019

a Measured in 1 wt% doped film in mCBP host.

b Measured in 1 wt% doped film in PMMA host.

c Measured in oxygen-free toluene solution (5 × 10^−5^M).

d Measured in 1 wt% doped film in DOBNA-OAr host.

^e^ΔE_ST_ = S_1_-T_1_.

f *Current efficiencies at luminance of maximum, 100 cd/m^2^*.

Further, it is found that the large conjugated DABNA-based material like ν-DABNA exhibited the highest blue TADF performances due to its small ΔE_ST_ and high PLQY. This result reflects the computational prediction by Pershin et al. (*vide supra*). So, the molecular design for the efficient blue MR-TADF should possess either large conjugated molecular structures or attachment of donor at the *para* position to the boron atom of the DABNA core.

## Conclusions and Outlook

We summarized the boron-containing aromatic acceptor moieties connected with several donors of push–pull small molecules for blue TADF OLED applications. The boron-based TADF materials exhibited good TADF performances with maximum EQE value as high as 38.15%, which is the highest value for blue TADF OLEDs reported so far. We found the order of type of boron-based materials to realize pure blue emission color, high PLQY, small ΔE_ST_, short delayed exciton lifetime, and high efficiency for unbridged < partially bridged < fully bridged boron compounds. Exclusively, the fully bridged oxygen-containing boron acceptor shows an additional property of horizontal dipole orientation which is desirable for highly efficient devices. On the other hand, the fully bridged nitrogen-containing boron materials (MR-TADF) show very narrow emission in deep blue region. It is concluded that the fully bridged boron compounds would be an ideal candidate for designing highly efficient deep blue TADF materials. Also, attachment of donor at the *para* position to the boron atom of MR-TADF materials would give deep blue color, small ΔE_ST_, high PLQY, and high efficiency. These results indicate that the boron materials are promising for the development of blue TADF OLEDs.

Though the boron-containing materials showed high efficiency, the lifetime of the blue TADF device is still a challenging task. As the delayed exciton lifetime of fully bridged boron materials is short, the usage of them as TADF assistant host for MR-TADF dopant materials in hyper-fluorescence devices has advantages of (i) an increase in color purity as emission originates from the MR-TADF dopant, (ii) the dramatically increased device efficiency due to the usage of both host and dopant as TADF materials, and (iii) the increased device lifetime due to the fast Forster resonance energy transfer from the TADF assistant host to the MR-TADF dopant.

## Author Contributions

HL, DK, and RL contributed equally to write and revise this manuscript. JR organized all the related tables and figures. JK supervised this work. All authors listed have made a substantial, direct and intellectual contribution to the work, and approved it for publication.

## Conflict of Interest

The authors declare that the research was conducted in the absence of any commercial or financial relationships that could be construed as a potential conflict of interest.

## References

[B1] Agou T. Kobayashi J. Kawashima T. (2006). Syntheses, structure, and optical properties of ladder-type fused azaborines. Org. Lett. 8, 2241–2244. 10.1021/ol060539n 16706496

[B2] Agou T. Kobayashi J. Kawashima T. (2007a). Development of a general route to periphery-functionalized azaborines and ladder-type azaborines by using common intermediates. Chem. Commun. 3204–3206. 10.1039/b706418g 17653389

[B3] Agou T. Kobayashi J. Kawashima T. (2007b). Electronic and optical properties of ladder-type heteraborins, Chem. A Eur. J. 13, 8051–8060. 10.1002/chem.200700622 17614309

[B4] Agou T. Matsuo K. Kawano R. Park I. S. Hosoya T. Fukumoto H. (2020). Pentacyclic ladder-heteraborin emitters exhibiting high-efficiency blue thermally activated delayed fluorescence with an ultrashort emission lifetime. ACS Mater. Lett. 2, 28–34. 10.1021/acsmaterialslett.9b00433

[B5] Ahn D. H. Kim S. W. Lee H. Ko I. J. Karthik D. Lee J. Y. (2019a). Highly efficient blue thermally activated delayed fluorescence emitters based on symmetrical and rigid oxygen-bridged boron acceptors. Nat. Photonics 13, 540–546. 10.1038/s41566-019-0415-5

[B6] Ahn D. H. Lee H. Kim S. W. Karthik D. Lee J. Jeong H.. (2019b). Highly twisted donor–acceptor boron emitter and high triplet host material for highly efficient blue thermally activated delayed fluorescent device. ACS Appl. Mater. Interfaces 11, 14909–14916. 10.1021/acsami.9b00931 30924634

[B7] Ahn D. H. Maeng J. H. Lee H. Yoo Y. Lampande R. Lee J. Y. (2020). Rigid oxygen-bridged boron-based blue thermally activated delayed fluorescence emitter for organic light emitting diode: approach towards satisfying high efficiency and long lifetime together. Adv. Optical Mater. 2000102. 10.1002/adom.202000102

[B8] Baldo M. A. O'brien D. F. You Y. Shoustikov A. Sibley S. Thompson M. E. (1998). Highly efficient phosphorescent emission from organic electroluminescent devices. Nature 395, 151–154. 10.1038/25954

[B9] Bonardi L. Kanaan H. Camerel F. Jolinat P. Retailleau P. Ziessel R. (2008). Fine-tuning of yellow or red photo- and electroluminescence of functional difluoro-boradiazaindacene films. Adv. Funct. Mater. 18, 401–413. 10.1002/adfm.200700697

[B10] Brown H. C. Dodson V. H. (1957). Studies in stereochemistry. XXII. the preparation and reactions of trimesitylborane. evidence for the non-localized nature of the odd electron in triarylborane radical ions and related free radicals1. J. Am. Chem. Soc. 79, 2302–2306. 10.1021/ja01566a076

[B11] D'aléo A. Sazzad M. H. Kim D. H. Choi E. Y. Wu J. W. Canard G.. (2017). Boron difluoride hemicurcuminoid as an efficient far red to near-infrared emitter: toward OLEDs and laser dyes. Chem. Commun. 53, 7003–7006. 10.1039/C7CC01786C 28513655

[B12] Elbing M. Bazan G. C. (2008). A new design strategy for organic optoelectronic materials by lateral boryl substitution. Angew. Chem. Int. Ed. 47, 834–838. 10.1002/anie.200703722 18081115

[B13] Entwistle C. D. Marder T. B. (2004). Applications of three-coordinate organoboron compounds and polymers in optoelectronics. Chem. Mater. 16, 4574–4585. 10.1021/cm0495717

[B14] Frath D. Massue J. Ulrich G. Ziessel R. (2014). Luminescent materials: locking π-conjugated and heterocyclic ligands with boron(III). Angew. Chem. Int. Ed. 53, 2290–2310. 10.1002/anie.201305554 24482312

[B15] Han S. H. Jeong J. H. Yoo J. W. Lee J. Y. (2019). Ideal blue thermally activated delayed fluorescence emission assisted by a thermally activated delayed fluorescence assistant dopant through a fast reverse intersystem crossing mediated cascade energy transfer process. J. Mater. Chem. C 7, 3082–3089. 10.1039/C8TC06575F

[B16] Hatakeyama T. Shiren K. Nakajima K. Nomura S. Nakatsuka S. Kinoshita K.. (2016). Ultrapure blue thermally activated delayed fluorescence molecules: efficient HOMO–LUMO separation by the multiple resonance effect. Adv. Mater. 28, 2777–2781. 10.1002/adma.201505491 26865384

[B17] Hirai H. Nakajima K. Nakatsuka S. Shiren K. Ni J. Nomura S.. (2015). One-step borylation of 1,3-diaryloxybenzenes towards efficient materials for organic light-emitting diodes. Angew. Chem. Int. Ed. 54, 13581–13585. 10.1002/anie.201506335 26380959

[B18] Im Y. Byun S. Y. Kim J. H. Lee D. R. Oh C. S. Yook K. S. (2017). Recent progress in high-efficiency blue-light-emitting materials for organic light-emitting diodes. Adv. Funct. Mater. 27:1603007 10.1002/adfm.201603007

[B19] Karthik D. Ahn D. H. Ryu J. H. Lee H. Maeng J. H. Lee J. Y. (2020). Highly efficient blue thermally activated delayed fluorescence organic light emitting diodes based on tercarbazole donor and boron acceptor dyads. J. Mater. Chem. C 8, 2272–2279. 10.1039/C9TC05950D

[B20] Kim K.-H. Kim J. J. (2018). Origin and control of orientation of phosphorescent and TADF dyes for high-efficiency OLEDs. Adv. Mater. 30:1705600. 10.1002/adma.201705600 29707823

[B21] Kim K. J. Kim G. H. Lampande R. Ahn D. H. Im J. B. Moon J. S. (2018). A new rigid diindolocarbazole donor moiety for high quantum efficiency thermally activated delayed fluorescence emitter. J. Mater. Chem. C 6, 1343–1348. 10.1039/C7TC04852A

[B22] Kitamoto Y. Namikawa T. Ikemizu D. Miyata Y. Suzuki T. Kita H. (2015). Light blue and green thermally activated delayed fluorescence from 10H-phenoxaborin-derivatives and their application to organic light-emitting diodes. J. Mater. Chem. C 3, 9122–9130. 10.1039/C5TC01380A

[B23] Kitamoto Y. Namikawa T. Suzuki T. Miyata Y. Kita H. Sato T. (2016). Dimesitylarylborane-based luminescent emitters exhibiting highly-efficient thermally activated delayed fluorescence for organic light-emitting diodes. Org. Electron. 34, 208–217. 10.1016/j.orgel.2016.04.030

[B24] Kondo Y. Yoshiura K. Kitera S. Nishi H. Oda S. Gotoh H. (2019). Narrowband deep-blue organic light-emitting diode featuring an organoboron-based emitter. Nat. Photonics 13, 678–682. 10.1038/s41566-019-0476-5

[B25] Lee Y. H. Park S. Oh J. Shin J. W. Jung J. Yoo S.. (2017). Rigidity-induced delayed fluorescence by ortho donor-appended triarylboron compounds: record-high efficiency in pure blue fluorescent organic light-emitting diodes. ACS Appl. Mater. Interfaces 9, 24035–24042. 10.1021/acsami.7b05615 28653832

[B26] Lee Y. H. Park S. Oh J. Woo S.-J. Kumar A. Kim J.-J. (2018). High-efficiency sky blue to ultradeep blue thermally activated delayed fluorescent diodes based on ortho-carbazole-appended triarylboron emitters: above 32% external quantum efficiency in blue devices. Adv. Opt. Mater. 6:1800385 10.1002/adom.201800385

[B27] Li G. Lou W. Wang D. Deng C. Zhang Q. (2019). Difluoroboron-enabled thermally activated delayed fluorescence. ACS Appl. Mater. Interfaces 11, 32209–32217. 10.1021/acsami.9b08107 31387348

[B28] Li P. Chan H. Lai S.-L. Ng M. Chan M-Y. Yam V. W-W. (2019). Four-coordinate boron emitters with tridentate chelating ligand for efficient and stable thermally activated delayed fluorescence organic light-emitting devices. Angew. Chem. Int. Ed. 58, 9088–9094. 10.1002/anie.201903332 31050130

[B29] Liang X. Tu Z-L. Zheng Y-X. (2019). Thermally activated delayed fluorescence materials: towards realization of high efficiency through strategic small molecular design. Chem A Eur. J. 25, 5623–5642. 10.1002/chem.201805952 30648301

[B30] Liang X. Yan Z-P. Han H-B. Wu Z-G. Zheng Y-X. Meng H.. (2018). Peripheral amplification of multi-resonance induced thermally activated delayed fluorescence for highly efficient OLEDs. Angew. Chem. Int. Ed. 57, 11316–11320. 10.1002/anie.201806323 29974588

[B31] Matsui K. Oda S. Yoshiura K. Nakajima K. Yasuda N. Hatakeyama T. (2018). One-shot multiple borylation toward BN-doped nanographenes. J. Am. Chem. Soc. 140, 1195–1198. 10.1021/jacs.7b10578 29120174

[B32] Matsuo K. Yasuda T. (2019). Boronate- and borinate-based π-systems for blue thermally activated delayed fluorescence materials. Chem. Commun. 55, 2501–2504. 10.1039/C8CC10282A 30741283

[B33] Mellerup S. K. Wang S. (2019). Boron-doped molecules for optoelectronics. Trends Chem. 1, 77–89. 10.1016/j.trechm.2019.01.003

[B34] Meng G. Chen X. Wang X. Wang N. Peng T. Wang S. (2019). Isomeric bright sky-blue TADF emitters based on bisacridine decorated DBNA: impact of donor locations on luminescent and electroluminescent properties. Adv. Opt. Mater. 7:1900130 10.1002/adom.201900130

[B35] Nguyen T. B. Nakanotani H. Hatakeyama T. Adachi C. (2020). The role of reverse intersystem crossing using a TADF-type acceptor molecule on the device stability of exciplex-based organic light-emitting diodes. Adv. Mater. 32:1906614. 10.1002/adma.201906614 31975459

[B36] Numata M. Yasuda T. Adachi C. (2015). High efficiency pure blue thermally activated delayed fluorescence molecules having 10H-phenoxaborin and acridan units. Chem. Commun. 51, 9443–9446. 10.1039/C5CC00307E 25959457

[B37] Oda S. Kawakami B. Kawasumi R. Okita R. Hatakeyama T. (2019). Multiple resonance effect-induced sky-blue thermally activated delayed fluorescence with a narrow emission band. Org. Lett. 21, 9311–9314. 10.1021/acs.orglett.9b03342 31613109

[B38] Park I. S. Matsuo K. Aizawa N. Yasuda T. (2018). High-performance dibenzoheteraborin-based thermally activated delayed fluorescence emitters: molecular architectonics for concurrently achieving narrowband emission and efficient triplet–singlet spin conversion. Adv. Funct. Mater. 28:1802031 10.1002/adfm.201802031

[B39] Park I. S. Numata M. Adachi C. Yasuda T. (2016). A phenazaborin-based high-efficiency blue delayed fluorescence material. Bull. Chem. Soc. Jpn. 89, 375–377. 10.1246/bcsj.20150399

[B40] Pershin A. Hall D. Lemaur V. Sancho-Garcia J-C. Muccioli L. Zysman-Colman E.. (2019). Highly emissive excitons with reduced exchange energy in thermally activated delayed fluorescent molecules. Nat. Commun. 10:597. 10.1038/s41467-019-08495-5 30723203 PMC6363735

[B41] Schellhammer K. S. Li T-Y. Zeika O. Körner C. Leo K. Ortmann F. (2017). Tuning near-infrared absorbing donor materials: a study of electronic, optical, and charge-transport properties of aza-BODIPYs. Chem. Mater. 29, 5525–5536. 10.1021/acs.chemmater.7b00653

[B42] Scholz S. Kondakov D. Lüssem B. Leo K. (2015). Degradation mechanisms and reactions in organic light-emitting devices. Chem. Rev. 115, 8449–8503. 10.1021/cr400704v 26230864

[B43] Song D. Yu Y. Yue L. Zhong D. Zhang Y. Yang X. (2019). Asymmetric thermally activated delayed fluorescence (TADF) emitters with 5,9-dioxa-13b-boranaphtho[3,2,1-de]anthracene (OBA) as the acceptor and highly efficient blue-emitting OLEDs. J. Mater. Chem. C 7, 11953–11963. 10.1039/C9TC04115J

[B44] Stachelek P. Alsimaree A. A. Alnoman R. B. Harriman A. Knight J. G. (2017). Thermally-activated, delayed fluorescence in O,B,O- and N,B,O-strapped boron dipyrromethene derivatives. J. Phys. Chem. A 121, 2096–2107. 10.1021/acs.jpca.6b11131 28245114

[B45] Suzuki K. Kubo S. Shizu K. Fukushima T. Wakamiya A. Murata Y.. (2015). Triarylboron-based fluorescent organic light-emitting diodes with external quantum efficiencies exceeding 20 %. Angew. Chem. Int. Ed. 54, 15231–15235. 10.1002/anie.201508270 26563845

[B46] Tang C. W. Vanslyke S. A. (1987). Organic electroluminescent diodes. Appl. Phys. Lett. 51, 913–915. 10.1063/1.98799

[B47] Turkoglu G. Cinar M. E. Ozturk T. (2017). Triarylborane-based materials for OLED applications. Molecules 22:1522. 10.3390/molecules22091522 28902157 PMC6151606

[B48] Uoyama H. Goushi K. Shizu K. Nomura H. Adachi C. (2012). Highly efficient organic light-emitting diodes from delayed fluorescence. Nature 492, 234–238. 10.1038/nature11687 23235877

[B49] Von Grotthuss E. John A. Kaese T. Wagner M. (2018). Doping polycyclic aromatics with boron for superior performance in materials science and catalysis. Asian J. Org. Chem. 7, 37–53. 10.1002/ajoc.201700495

[B50] Yamaguchi S. Wakamiya A. (2006). Boron as a key component for new π-electron materials. Pure Appl. Chem. 78, 1413–1424. 10.1351/pac200678071413

[B51] Yang X. Guo H. Liu B. Zhao J. Zhou G. Wu Z. (2018). Diarylboron-based asymmetric red-emitting Ir(III) complex for solution-processed phosphorescent organic light-emitting diode with external quantum efficiency above 28%. Adv. Sci. 5:1701067 10.1002/advs.201800950 PMC5979779 29876224

[B52] Zampetti A. Minotto A. Squeo B. M. Gregoriou V. G. Allard S. Scherf U.. (2017). Highly efficient solid-state near-infrared organic light-emitting diodes incorporating A-D-A dyes based on α,β-unsubstituted BODIPY moieties. Sci. Rep. 7:1611. 10.1038/s41598-017-01785-2 28487525 PMC5431651

[B53] Zhang Y. Zhang D. Wei J. Liu Z. Lu Y. Duan L. (2019). Multi-resonance induced thermally activated delayed fluorophores for narrowband green OLEDs. Angew. Chem. Int. Ed. 58, 16912–16917. 10.1002/anie.201911266 31553115

